# GlucoSTRESS - A project to optimize glycemic control in a level C (III) Portuguese intensive care unit

**DOI:** 10.5935/0103-507X.20210015

**Published:** 2021

**Authors:** Ana Catarina Emidio, Rita Faria, Bruno Bispo, Vítor Vaz-Pinto, António Messias, Carlos Meneses-Oliveira

**Affiliations:** 1 Internal Medicine Service, Centro Hospitalar de Setúbal - Setúbal, Portugal.; 2 Intensive Care Service, Hospital Beatriz Ângelo - Loures, Lisboa, Portugal.

**Keywords:** Hyperglycemia, Hypoglycemia, Diabetes mellitus, Insulin, Glycemic index, Intensive care units, Hiperglicemia, Hipoglicemia, Insulina, Diabete mellitus, Índice glicêmico, Unidades de terapia intensiva

## Abstract

**Objective:**

To double the percentage of time within the 100 - 180mg/dL blood glucose range in the first three months following a phased implementation of a formal education program, and then, of an insulin therapy protocol, without entailing an increased incidence of hypoglycemia.

**Methods:**

The pre-intervention glycemic control was assessed retrospectively. Next, were carried out the implementation of a formal education program, distribution of manual algorithms for intravenous insulin therapy - optimized by the users, based on the modified Yale protocol - and informal training of the nursing staff. The use of electronic blood glucose control systems was supported, and the results were recorded prospectively.

**Results:**

The first phase of the program (formal education) lead to improvement of the time within the euglycemic interval (28% to 37%). In the second phase, euglycemia was achieved 66% of the time, and the incidence of hypoglycemia was decreased. The percentage of patients on intravenous insulin infusion at 48 hours from admission increased from 6% to 35%.

**Conclusion:**

The phased implementation of a formal education program, fostering the use of electronic insulin therapy protocols and dynamic manuals, received good adherence and has shown to be safe and effective for blood glucose control in critically ill patients, with a concomitant decrease in hypoglycemia.

## Abstract (pt)

**Objetivo:**

Duplicar a percentagem de tempo no intervalo glicêmico 100 - 180mg/dL nos primeiros 3 meses após implementação faseada de um programa de educação formal e, posteriormente, de um protocolo de insulinoterapia, sem condicionar um aumento da frequência de hipoglicemia.

**Métodos:**

Foi feita a avaliação retrospetiva do controle glicêmico pré-intervenção. Foram realizados: implementação de um programa formal de educação; distribuição de algoritmos manuais de insulinoterapia endovenosa - otimizados pelos utilizadores, a partir do protocolo de Yale modificado - e formação informal da equipe de enfermagem. Foi dado apoio à utilização dos sistemas eletrônicos de controle glicêmico e do registo prospetivo dos resultados.

**Resultados:**

A primeira fase do programa (educação formal) melhorou o tempo no intervalo euglicêmico (28% para 37%). A segunda fase permitiu atingir 66% do tempo de euglicemia, com diminuição das hipoglicemias. A percentagem de doentes sob perfusão endovenosa de insulina às 48 horas de internamento aumentou (6% para 35%).

**Conclusão:**

A implementação faseada de um programa formal de educação que favoreceu a aplicação de protocolos de insulinoterapia eletrônicos e manuais dinâmicos demonstrou ter aderência e ser segura e eficaz no controle glicêmico no doente crítico, com diminuição concomitante das hipoglicemias.

## INTRODUCTION

Hyperglycemia is frequent in critically ill patients and is observed in up to 50% of the patients within the first 48 hours following admission to the intensive care unit (ICU). It affects both diabetic and non-diabetic patients.^(1,2)^

Although stress hyperglycemia was initially considered to be associated with pre-diabetic individuals, later it was discovered to occur also in patients with normal glucose tolerance and is resolved upon recovering from the acute condition.^(2-4)^ It results from multiple factors, including increased cortisol, catecholamines, glucagon, growth hormone, tumor necrosis factor-alpha (TNF-α), and interleukin (IL) 1 and IL-6.^(5)^ These factors promote increased glucogenesis, glycogenolysis, and insulin resistance - especially hepatic insulin resistance. This is the case in up to 80% of critically ill patients.^(6)^

When uncontrolled, hyperglycemia in critically ill patients is associated with a worse prognosis, both in terms of morbidity and mortality; therefore, it is extremely important to prevent and control hyperglycemia.^(7)^ Stress hyperglycemia is associated with longer hospital stays, higher infection rates, higher resource consumption, increased risk of complications and mortality. Therefore, these patients should be identified as early as possible.^(8)^ Hypoglycemia is also known to be associated with increased mortality. Together with blood glucose variability (coefficient of variation > 20%), it is a major determinant of an appropriate blood glucose control.^(9-11)^

The independent association between hyperglycemia and mortality for non-diabetics is robust, however, not so for diabetes *mellitus* patients.^(12)^ This suggests that diabetic patients may not benefit from such strict glycemic control.^(2,5,12,13)^

More recently, the concept of time in range has emerged, which is a measure of how long blood glucose is kept within a certain range. This is suitable for assessing the safety and efficacy of blood glucose control and can be considered a dysglycemia severity marker and a quality-of-care indicator.^(12,14)^ Higher times in range (> 80%) have been associated with reduced ICU mortality and length of stay, regardless of the underlying condition severity.^(12,14)^ Its easy calculation can make it a useful tool for ICU monitoring, although in clinical practice, achieving > 80% times in range is a real challenge.^(12)^

The use of validated insulin therapy protocols allows maintaining blood glucose levels within the target, reducing hypoglycemia and hyperglycemia episodes, with less variability.^(15)^ When compared with manual protocols, electronic protocols have shown higher adherence and improved glycemic control, with a lower risk of hypoglycemia.^(15-17)^ Among the manual protocols, the best control was achieved using dynamic algorithms, that account for blood glucose variations and provide personalized feedback of the insulin response.^(18)^

The optimal glycemic range in the critically ill patient is still controversial, but some guidelines, including those from the American Diabetes Association (ADA), consider persistent hyperglycemia (above 180mg/dL) a suitable trigger for insulin therapy. Blood glucose should be kept within 140 - 180mg/dL. A lower target may be established, provided it is achievable without increasing the risk of hypoglycemia.^(8,13,19-21)^

Sliding-scale insulin regimens do not effectively prevent hypoglycemia and are associated with greater glycemic variability and are not recommended for critically ill patients.^(13,17)^ For insulin therapy in the ICU, intravenous (IV) infusion should be preferred. Allowing a rapid onset of action and short duration, this route provides better dose titration, adapting to rapid blood glucose changes occurring in these patients, therefore preventing insulin absorption to be affected by peripheral vasoconstriction.^(17)^

Recognizing the importance of the time on blood glucose target, as well as the use of insulin protocols for critically ill patients, we discuss the implementation of a formal education program fostering the use of electronic insulin therapy protocols (Space Glucose Control^®^) and dynamic manuals in a C (III) level Portuguese ICU. This work aimed to double the percentage of time within the target range 100 - 180mg/dL in the first three months after the implementation, without increasing the incidence of hypoglycemia (0 severe < 40mg/dL, and < 3 moderate 41 - 70mg/dL), as well to determine the program's efficacy and safety.

## METHODS

The Intensive Care Service of the *Hospital Beatriz Ângelo* is a mixed medical and surgical unit with 22 beds, from which 10 are level III; annually around 2,000 patients are admitted. The medical staff consists of intensivists and Internal Medicine physicians in Intensive Care Medicine training. It also includes training interns from other specialties. In level III, the nurse-patient ratio is usually 2:1 and may reach 1:1, depending on the workload. This study was approved by the Ethics Committee of the *Hospital Beatriz Ângelo*, reference number 3134/2019_MJHNO.

The perception by the ICU's healthcare professionals of an inadequate blood glucose control motivated us to carry out a retrospective assessment of data on the time patients were kept in the glycemic range of 100 - 180mg/dL, the incidence of hypoglycemia, and the percentage of patients without prescribed insulin therapy or IV insulin infusion at 48 hours from admission. Based on this retrospective evaluation, the effectiveness of the usual care was established.

Based on these data, a project aimed at optimization of blood glucose control in the ICU was therefore designed and implemented. As compared to a protocol, this project had a dynamic character and was updated according to the users' feedback. Furthermore, it allowed for unforeseen results, enabled adaptation to the needs, and included a protocol implementation. Consequently, the adherence was expected to be higher. The formal work team included three medical doctors and two nurses.

**Figure 1:**
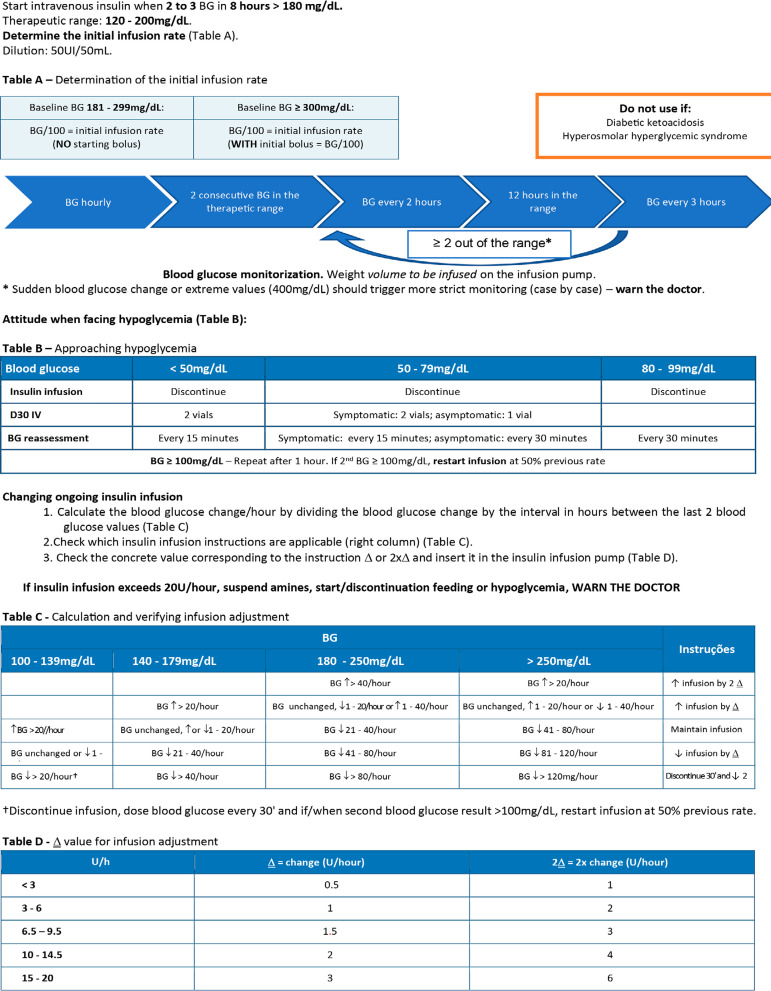
Insulin therapy schedule adapted from the modified Yale protocol.

The first phase consisted of the implementation of a formal education program involving more than 80% of the medical staff and nursing team leaders. Formative 30-minute sessions were held, aimed at raise awareness of blood glucose control in critically ill patients. Subsequently, an informal adherence stimulation program was implemented, consisting of real-time blood glucose control and publication of results. Manual IV insulin therapy algorithms adapted from the modified Yale protocol were also distributed (Figure 1). To involve a larger number of nurses, bedside training was provided to the nurse responsible for the patient, who would subsequently be responsible for training the next shift's nurse. Simultaneously, the alternative use of an electronic blood glucose control protocol was stimulated (Space Glucose Control^®^). During the algorithm implementation, daily sessions were held with all the nursing staff on duty that day, to review the procedure and clarify doubts.

BC - blood glucose.

Source: Modified and adapted from Ngalob Q, Jimeno C, Isip-Tan IT. Evaluation of effectiveness and safety of an ICU insulin infusion protocol. J Asean Fed Endocr Soc. 2014;29(1):33-41.

The manual schedule (Figure 1) considered a blood glucose target of 140 - 180mg/dL and should not be used in patients with diabetic ketoacidosis or hyperosmolar hyperglycemic syndrome. The more comprehensive "therapeutic range" concept was introduced, with the sole purpose to guide monitoring and ease the nursing staff workload. The scheme provides clear indications on when and how to be started, allowing the nursing team autonomy. It involves the blood glucose determination, choosing one of the four columns in Figure 1 - Table C. Subsequently, the blood glucose variation per hour should be calculated by subtracting the current value from the previous one. After this calculation, correspondence must be sought with the column where the perfusion adjustment instructions are (Figure 1 - Table D). This protocol also provides information on how to act when facing hypoglycemia (Figure 1 - Table B) and when the doctor should be called.

It was recommended to start insulin infusion according to the protocol (either manual or electronic) upon identification of two out of three blood glucose values above 180mg/dL in 8 hours. The decision of starting the scheme was always up to the professional.

To recall some concepts, daily sessions of about 10 minutes were held with the nursing staff, allowing doubts to be clarified. At the end of the project, the results were presented to the whole team.

Data were prospectively collected in three different phases: one month (after the education program), three months (after the protocol availability), and six months after the project start. The inclusion clinical variables included: age, gender, previous diagnosis of diabetes, the main reason for admission to the ICU (medical/surgical), and the Acute Physiology and Chronic Health Evaluation (APACHE) II score. Stress hyperglycemia was diagnosed by a less 6.5% glycated hemoglobin (HbA1c), as dosed within the first 72 hours from the admission. Patients staying in the ICU for less than 24 hours, with diabetic ketoacidosis, or hyperosmolar hyperglycemic syndrome, were excluded.

Blood glucose values and information on the insulin regimen were retrieved from the medical records (Soarian^®^) Clinicals) or the nursing records (Innovian^®^). Blood glucose values were obtained with Abbott's Freestyle Precision NEO H glucometer (capillary or arterial blood) or from arterial blood gas tests. The frequency was determined by the protocol. When blood glucose was not hourly assessed, the values in the hours before and after the missing value were averaged, making it possible to consider all patients equally in the percentage calculation.

The Microsoft Excel^®^ software was used for data recording and processing. The results are presented as mean plus/minus standard deviation or as a percentage.

## RESULTS

### Before the project

To assess the glycemic control achieved with previous ICU strategies, the percentage of time in the 100 - 180mg/dL range for patients with insulin infusion and with no prescribed insulin regimen at 48 hours after admission, were determined. Moderate and severe hypoglycemias were also recorded.

The characteristics of patients are shown in table 1. For one month, all patients staying more than 24 hours and showing at least two blood glucose values above 180mg/dL were included. Of the 15 patients, mean age 70.8 ± 11.4 years, most were male and had a medical admission. In 67% of the cases, the patient had been previously diagnosed with diabetes *mellitus*. The average APACHE II score was 22.6 ± 8.5.

With the previous strategies, the percentage of time in the 100 - 180mg/dL range was 28%. At 48 hours from admission, only one patient was on IV insulin therapy and one-third of the patients had no prescribed insulin regimen. Space Glucose Control^®^ had not been used in any patient. Six moderate hypoglycemias (40 - 70mg/dL) were identified.

### Project - First month

After implementing the formal education program the results were evaluated to determine its effectiveness. During this period 16 patients were identified, most of them male, mean age of 72.9 ± 10.8 years. Admissions were mostly for medical conditions. Most of the patients (57%) were diabetic, and one patient was newly diagnosed with Diabetes mellitus. The mean APACHE II score was 23.1 ± 7.6 (Table 1).

**Table 1:** Characterization of the patients

	Before the project n = 15	First month n = 16	Third month n = 29
Gender			
Female	6 (40)	6 (37)	12 (41)
Male	9 (60)	10 (63)	17 (59)
Age	70.8 ± 11.4	72.9 ± 10.8	69.4 ± 12.9
Reason for admission			
Medical	8 (53)	10 (63)	15 (52)
Surgical	7 (47)	6 (37)	14 (48)
*Diabetes mellitus*			
Known	10 (67)	9 (57)	14 (48)
Not previously known	0 (0)	1 (6)	2 (7)
Stress hyperglycemia	5 (34)	6 (37)	13 (45)
APACHE II	22,6 ± 8,5	23,1 ± 7,6	19,9 ± 10,2

APACHE - Acute Physiology and Chronic Health Evaluation. Results expressed as n (%) or mean ± standard deviation.

Compared to the previous period, table 2 shows an increase in the percentage of patients in the glycemic target (37%). The percentage of patients on IV insulin therapy increased to 19%, and Space Glucose Control^®^ was used in one patient. Despite the improvement in starting IV insulin therapy, in 25% of the patients, no insulin therapy regimen was prescribed at 48 hours from admission. During this period three moderate hypoglycemias were identified.

**Table 2:** Evaluation of the glycemic control

	Before the project n = 15	First month n = 16	Third month n = 29
Time in the target range (100 - 180mg/dL)	28.0	37.0	66.0
Patients with IV insulin at 48 hours	1 (6)	3 (19)	10 (35)
Patients with no insulin regimen at 48 hours	5 (33)	4 (25)	0 (0)
Patients on Glucose Space Control®	0 (0)	1 (6)	3 (10)
Hypoglycemia			
Moderate	6	3	1
Severe	0	0	0

IV - intravenous.

### Project - Third month

During the second month, the insulin therapy plans were tested and distributed. The first version was changed to incorporate suggestions from the nursing staff aimed at easing the workload. These changes were mainly related to monitorization. By the third month, the results were evaluated.

Of the 29 patients, mean age of 69.4 ± 12.9 years, 59% were male. The reason for admission was medical in 52% of the patients. Two previously unknown diabetes *mellitus* patients were identified, and only 48% of the patients were previously diagnosed with Diabetes *mellitus*. The remainder were stress hyperglycemia patients. The mean APACHE II score was 19.9 ± 10.2 (Table 1).

The insulin therapy protocol entailed a 66% increase in the percentage of time within the 100 - 180mg/dL range. At 48 hours after admission, more than one-third of the patients had insulin infusion, and all patients had a prescribed insulin regimen. During this period, the incidence of hypoglycemia was substantially reduced, with only one easily reversed case of moderate hypoglycemia identified (Table 2).

## DISCUSSION

Currently, the more conservative recommendations for blood glucose targets in critically ill patients point to values between 140 and 180mg/dL. As long as the absence of hypoglycemia is assured, lower values may be achieved.^(8,13,19-21)^ Therefore, the targeted blood glucose level for our ICU was established within the 100 - 180mg/dL range, adapting the algorithm to keep blood glucose values within this range, to preserve safety.

Achieving high percentages of time in the glycemic range demands commitment of time and the entire team's availability. The increased workload associated with insulin therapy protocols falls mostly on the nursing staff and the time spent in tasks related to blood glucose control with one single patient may take as high as 2 hours.^(22)^ However, in a study in 2005^(23)^ it was shown that, after understanding the relevance of glycemic control, nurses were more willing to comply with insulin therapy protocols.

The project's first phase consisted of a formal education program involving most of the ICU medical staff and nursing leaders. Providing the same information for both medical doctors and nurses has been shown to improve blood glucose control strategies.^(24,25)^ Indeed, following the formative sessions, increased awareness of hyperglycemia was observed; this translated into results achieved during the first month when the time in the 100 - 180mg/dL blood glucose range was increased by almost 10%. The increasing commitment to blood glucose control can also be seen in the substantially increased number of patients with IV insulin therapy regimens at 48 hours from admission. Also, the effort to overcome the previous bad experience with electronic protocols (Space Glucose Control^®^) resulted in its use in one patient.

The literature reports these devices to bring benefits; therefore we assessed the reasons why past bad experiences with electronic protocols were reported.^(15,16,22,26)^ The reasons were mostly related to the increased workload associated with more frequent measurements and difficulty in maintaining glycemic stability. It was found that the ICU had no standard for performing blood glucose measurements; the use of glucometers in critically ill patients entails reduced accuracy, as these patients have often conditions reducing the peripheral perfusion, such as shock, vasopressors, edema, hypotension, etc.^(19,27)^ As a mathematical algorithm, the inconsistency between glucometer values and those from the arterial blood gas analysis, may contribute to reducing the glycemic stability.

To eliminate methodological variability, using the same method during the entire hospitalization was recommended.

In addition to monitoring issues, for the proper functioning of electronic algorithms, the list of drugs included in the device should be frequently reviewed. In this way, a discrepancy between the effectively given carbohydrates and the amount considered by the algorithm can be avoided. By the time when this study was being conducted this update had not yet been done, causing calculation inconsistencies and values out of the therapeutic range.

During the second phase of the project, insulin therapy algorithms were distributed. A testing period was allowed so the entire medical and nursing teams could use and analyze them, and suggest changes.

Most requested changes were related to the glycemic monitorization frequency, and the formal team chose to introduce the therapeutic range concept, aimed at guiding monitorization. This concept effectively reduced the monitorization frequency, consequently easing the workload, therefore improving the initial implementation phase acceptance. The increased nursing workload is described in the literature as the most important limitation for the use of insulin therapy protocols.^(18,22)^ Especially in the early phases, reducing the frequency of monitorization may compromise glycemic stability, with an inconsistent control of hypoglycemia and risk of undetected hypoglycemia.^(22)^ This may have affected the final percentage of time in the range, however without increasing hypoglycemias.

Another often described fear is that in the daily routine of the ICU, it is not possible respecting the monitoring schedules and a monitoring time can be easily missed.^(22,24,25)^ It was suggested to set a given time in the infusion pump in the volume to be infused mode so that a beep sound would alert the nurse.

After the trial period, the program was implemented as a protocol. The use of nursing team-initiated protocols is associated with better outcomes.^(18,25)^ Indeed, after the protocol implementation, a significant increase of patients receiving insulin infusion during hospitalization was observed (35% with the dynamic manual protocol and 10% with the electronic protocol). It was also observed that all patients had a prescribed insulin regimen 48 hours after admission. Besides, providing clear instructions on how to act in face of hypoglycemia and conditions that should require medical attention contributed to better compliance.^(25)^

Defining the 140 - 180mg/dL range as a target for the insulin therapy regimen allowed us to achieve the study objective: 66% of the time in the range of 100 - 180mg/dL, and to decreased incidence of hypoglycemia. These data agree with other protocols with less strict targets.^(28)^

Team motivation is a key success factor for the implementation of blood glucose control improvement projects.^(22)^ Therefore, during all phases, the results were publicized and posted on the ICU. At the end of the third month, the results were presented to the entire team, launching the next audits' goals.

The main limitations of this study are the reduced number of patients and the study duration. Additionally, for patients who did not have hourly blood glucose measurements, we averaged the before and after values. This was necessary so that all included patients would contribute equally to the percentage calculation.

As a strength, the project culminated in the implementation of an insulin therapy protocol (target 140 - 180mg/dL) that provides autonomy to the nursing team and allows achieving the glycemic range without compromising safety. More frequent monitoring may improve the percentage of time in the range and safety.

## CONCLUSION

The GlucoSTRESS consisted of a clinical quality improvement project, aimed at testing the feasibility, efficacy, and safety of glycemic control promoting measures. The use of a retrospective control allowed us to assess the effectiveness of the previous strategies and to identify a baseline level of control from which the project should start.

The present work demonstrates the effectiveness and safety of the phased implementation of a formal education program fostering the use of electronic (Space Glucose Control^®^) and dynamic manual insulin therapy protocols. The continuation of the formal education program, with frequent audits and presentation of results, is expected to improve glycemic control and the insulin therapy algorithm.
